# Illness Anxiety Disorder Manifesting as Huntington's Disease: A Rare Case Report

**DOI:** 10.1002/ccr3.72916

**Published:** 2026-06-09

**Authors:** Amin Ziyaei, Aliakbar Marzban, Fatemeh Pourjafar, Mohammad Sadegh Khooshab, Reza Bidaki

**Affiliations:** ^1^ Student Research Committee Shahid Sadoughi University of Medical Sciences Yazd Iran; ^2^ Department of Psychiatry, Research Development Center, Shahid Sadoughi Hospital, School of Medicine Shahid Sadoughi University of Medical Sciences Yazd Iran; ^3^ Department of Psychology, Yazd Branch Islamic Azad University Yazd Iran; ^4^ Department of Psychiatry, Research Center of Addiction and Behavioral Sciences, Non‐Communicable Diseases Research Institute Shahid Sadoughi University of Medical Sciences Yazd Iran

**Keywords:** anxiety, Huntington disease, hypochondriasis, illness behavior, psychiatry

## Abstract

Illness anxiety disorder (IAD) is a psychological disorder characterized by an exaggerated preoccupation with the possibility of having a serious illness even in the absence of somatic symptoms. A 38‐year‐old woman presented to the clinic complaining of rhythmic and involuntary movements of her head and body. She had a history of chronic stress and recurrent significant loss, and she grew up in a highly emotional family. She remained preoccupied with the possibility of Huntington's disease, despite unremarkable neurological and laboratory findings. In the past, there have been some reports about Illness anxiety disorder (IAD) presenting with neurodegenerative diseases, particularly multiple sclerosis. However, this case is unique because the patient has shown severe anxiety due to the belief that they have Huntington's disease, which is a rare occurrence.

## Key Clinical Message

Illness anxiety disorder typically manifests as fear of common disorders like cancer, but in rare cases, it may present as movement disorders like Huntington's disease. The patient's previous psychological conditions, especially anxiety, are indicative. Explaining the illness, proper examinations, and preventing repeated clinical examinations are aspects of treating the condition.

## 1 Introduction

Illness Anxiety Disorder (IAD), previously referred to as hypochondriasis, is a mental health disorder that consists of anxiety around having or developing a serious illness [1]. Individuals with IAD may devote time to thinking about their health, even when no medical evidence of illness or health problems exists. They will be preoccupied with worry and may make compulsive or excessive efforts, such as checking their body for signs of ill health or asking health care providers for verbal reassurance about their health status [2, 3]. With an equal prevalence in both sexes, this illness is chronic and diverse [4].

There are two clinical manifestations of IAD: Care‐seeking type: Because of their worries, the person regularly seeks medical help. Care‐avoidant type: Despite their concerns, the person hardly ever seeks medical attention [2]. The available epidemiological data are limited because of recent reclassifications. This disorder seems to affect older age groups more than younger ones [3, 5, 6, 7].

These people have a strong belief that they have a serious illness, even in the face of negative lab results and unimpressive physical examinations. This leads to anxiety and distress, which can then show up as symptoms in the person, creating a vicious cycle [2]. Previously, there has been a report of a mistake in distinguishing between Huntington's disease and conversion disorder [8]. The study's case study is significant because the patient has an overvalued idea that he is afflicted with Huntington's disease, which is a rare occurrence in illness anxiety disorder.

## 2 Case History/Examination

A 38‐year‐old right‐handed woman with a high school education and 10 years of marriage presented to the clinic. She complained of involuntary and rhythmic movements of her head and body, particularly noticeable in a semi‐standing position. Additionally, she reported sudden jerks in her fingers when bringing them together or separating them. She also experienced a sensation of internal shock in her head and sudden jerks during sleep. In the last year, she had believed that she had Parkinson's disease.

In the study of patient family history, both her uncle and aunt were initially diagnosed with Parkinson's disease in their 30s to 40s, which was later reclassified as Huntington's disease. She progressed through the normal developmental stages during childhood and grew up in a family of nine as the eldest child. The patient and other family members mentioned diagnostic criteria for anxiety disorder.

In her psychiatric history, she had experienced recurrent significant loss in recent years: the loss of her father and, later, her 17‐year‐old brother. In the first 6 years of her marriage, she had no children, which exacerbated her anxiety and worry. After the birth of her first child, she experienced postpartum depression, feeling that her life had lost all meaning. During her second pregnancy, she developed severe obsessive‐compulsive symptoms. At 6 months of pregnancy, she underwent CT angiography ordered by a specialist due to suspected pulmonary embolism, which ruled out the diagnosis. Further investigations led to a diagnosis of a panic attack. However, after reading various articles about the risks of CT angiography to the fetus, her anxiety worsened, and she began attending regular checkups. After the birth of her second child, her obsessive thoughts about the baby's health persisted.

Overall, in assessing the patient's psychiatric evaluation, the patient was an individual suffering from obsessive thoughts, anxiety, and dependent personality disorder, with marital and family problems and pathological grief related to the loss of close family members as a comorbidity.

## 3 Methods (Differential Diagnosis, Investigations, and Treatment)

Given the possibility of the patient having a movement disorder, neurological tests were ordered. In these circumstances, in addition to Huntington's disease, the diagnosis of other movement disorders is also considered, especially Huntington's disease‐like syndromes [9].

All physical and neurological examinations (Table 1) were normal, and there was no evidence supporting Huntington's disease, Huntington's disease‐like syndromes, or any other movement disorders. She exhibited only a postural tremor, which appeared to be voluntarily exaggerated.

**TABLE 1:** Neurological examination findings.

Examinations	Results
Athetoid movements	Negative
Motor Persistence	Normal
Gait change	Negative
Myerson test	Normal
Bradykinesia	Negative
Shuffling gait	Negative
Rigidity	Negative
Resting tremor	Negative
Finger tap test	Normal

The patient's condition indicated the absence of a neurodegenerative disease. She exhibited a series of tremors and occasionally chorea‐like movements (mostly in the thumb of the left hand). Still, these movements were exacerbated by anxiety and stress, decreased with distraction, and do not possess the characteristics required for a chorea diagnosis. She had a relative who suffered from movement disorders for some time and subsequently passed away. Given the patient's past psychological history, she was highly suggestible. After hearing that the cause of death of that individual was Huntington's disease, she searched online and experienced significant perceptual and cognitive errors, giving them undue importance. She compares herself to that patient and believes her condition could develop into chorea.

The absence of reliable first‐degree family history in the patient, the patient's age not aligning with Huntington's disease, the mismatch of movements with chorea‐like movements, the fluctuation of movements which increased with attention and decreased with distraction, the lack of motor perseveration, and the patient's visit a year ago expressing fear of developing Parkinson's disease suggested a psychological rather than a neurological nature to the illness. Furthermore, given the absence of symptoms related to Huntington's disease and the lack of a reliable family history of the disease among close relatives, there was no need for genetic testing.

Drug‐induced movement disorders could represent another possible explanation for the patient's tremor and involuntary movements [10]. These disorders are typically associated with the use of psychiatric medications and may present in either acute or chronic forms with various clinical manifestations. In addition, movement disorders secondary to illicit substance use, such as Amphetamines, should also be considered in the differential diagnosis. However, a comprehensive and reliable patient history denying medication or illicit drug use excluded these diagnoses.

For these patients, a diagnosis of factitious disorder with primary gain was considered. Factitious disorder (previously Munchausen syndrome) is a psychological disorder in which a person tries to seek medical attention by feigning clinical symptoms. Typically, these patients seek attention [11]; However, in our case study, the patient was not seeking attention but had a genuine concern about the illness. Furthermore, the absence of primary gain in the form of attention, sympathy, or physical care reduces the likelihood of this diagnosis.

Furthermore, malingering was also a possibility, but given the lack of identified secondary gain, like receiving payment from an insurance company or receiving disability benefits, the diagnosis of malingering was unlikely. Therefore, she was more likely to have an overvalued ideation, and based on the patient's history, the diagnosis leaned towards illness anxiety disorder, which was additionally confirmed by DSM‐5 diagnostic criteria.

## 4 Conclusion and Results (Outcome and Follow‐Up)

The patient was examined by a neuropsychiatrist every 1 to 2 months for 6 months; at each visit, the patient underwent neurological and psychological assessments. She was reassured that their problem was not unsolvable, which was done by explaining Huntington's disease and its characteristics, as well as illness anxiety disorder. She was advised to avoid excessive visits to physicians and unnecessary tests and imaging. Treatment of the patient's underlying anxiety and depression was also carried out using antidepressants (SSRIs) to manage the patient's depression and beta‐blockers to reduce the patient's sympathetic tone, which led to a reduction in the symptoms of the illness, including tremor frequency. Additionally, the patient stopped repeated physician visits and had no recurrence during follow‐up.

## 5 Discussion

While visiting a physician, patients with illness anxiety disorder (IAD) believe that they are suffering from a disease and continue to seek to refute their doctor's diagnosis even after being declared healthy, citing reasons such as not paying attention to symptoms or not taking their illness seriously [12]. The patient, even though specialist physicians had ruled out a diagnosis of Huntington's disease after examinations, had a strong claim of suffering from the illness. Based on the neuropsychiatrist's examination, she did not exhibit symptoms of motor impersistence, choreiform movement, or a family history and genetic background of Huntington's disease; the only observed symptom was a fine postural tremor. Additionally, there was a significant and exaggerated concern regarding the tremor and the possibility of Huntington's disease. Overall, the diagnosis of Huntington's disease seemed unlikely; therefore, attention was directed towards behavioral disturbances.

One reason for this belief could be health‐related cognitive distortions shaped by family experiences [13]. The patient had cognitive errors regarding the illness of a distant relative, who was initially said to have Parkinson's disease and subsequently became disabled and passed away. After a year, the diagnosis changed to Huntington's disease. In this situation, our patient had come to believe that they were afflicted with Huntington's disease. In contrast, initially, before the secondary diagnosis of their relative, the patient thought that she had Parkinson's disease. The patient was experiencing severe anxiety and worry, had family issues with their husband related to insufficient support during the illness, and appeared to be highly “suggestible”. There was no secondary gain, so the diagnosis of malingering did not apply. Additionally, the patient had a bit of attention‐seeking behavior. Furthermore, we predicted that if another family member of the patient were to develop a rare or severe disease, such as cancer, the patient would acknowledge having this illness and return to the neuropsychiatrist. There have been reports of patients presenting with beliefs of having diseases such as rabies and pulmonary thromboembolism [4, 14], or other neurodegenerative diseases like multiple sclerosis. However, this is a rare case of this disease, with the belief of having Huntington's disease.

The patient's treatment began with the use of anti‐anxiety and antidepressant medications (SSRIs and anxiolytics). In addition, efforts are made to reassure the patient regarding their health, to prevent unnecessary paraclinical tests, and to divert their mind from thoughts about the illness.

Illness anxiety disorder can rarely manifest as a belief in having a neurodegenerative disease, such as Huntington's disease. The importance of this is that a thorough examination of the patient for movement issues, combined with consideration of the patient's family history, can prevent unnecessary tests and paraclinical measures. The patient can be assured that this is not a physical illness, but rather an anxiety disorder, and that it can be managed by managing the patient's stress and anxiety.

## Author Contributions

**Amin Ziyaei:** writing – original draft, writing – review and editing. **Aliakbar Marzban:** writing – original draft, writing – review and editing. **Fatemeh Pourjafar:** data curation, investigation. **Mohammad Sadegh Khooshab:** writing – original draft, writing – review and editing. **Reza Bidaki:** conceptualization, supervision, writing – review and editing.

## Funding

The authors have nothing to report.

## Ethics Statement

This study was approved by the Ethics Committee of Shahid Sadoughi University of Medical Sciences, Yazd, Iran (Approval code: IR.SSU.MEDICINE.REC.1404.081).

## Consent

The patient has signed a consent form and allowed this article to be published.

## Conflicts of Interest

The authors declare no conflicts of interest.

## Data Availability

The data that support the findings of this study are available from the corresponding author upon reasonable request.
